# Medical Books of Lindsay & Blakiston

**Published:** 1873-05

**Authors:** 


Medical Books.—We would call the attention of our readers to the catalogue of Messrs. Lindsay & Blakiston, contained in the present number This list comprises many eminently practical works. It will be seen that this house have made arrangements with the Messrs. Churchill, of London, whereby they are prepared to furnish their publications at reduced rates. Many of the works published by Lindsay & Blakiston have taken their place as standard publications.

